# Prevalence of Active Trachoma and Associated Factors in Areka Town, South Ethiopia, 2018

**DOI:** 10.1155/2020/8635191

**Published:** 2020-10-16

**Authors:** Melese Menta Alambo, Eyasu Alam Lake, Shimelash Bitew Workie, Addisu Yeshambel Wassie

**Affiliations:** ^1^Department of Public Health, College of Health Science and Medicine, Wolaita Sodo University, Wolaita Sodo, Ethiopia; ^2^Department of Nursing, College of Health Science and Medicine, Wolaita Sodo University, Wolaita Sodo, Ethiopia; ^3^Department of Midwifery, College of Health Science and Medicine, Wolaita Sodo University, Wolaita Sodo, Ethiopia

## Abstract

**Background:**

Globally, 1.2 billion people live in trachoma endemic areas, 40.6 million people are suffering from active trachoma, and 48.5% of the global burden of active trachoma is distributed in five countries including Ethiopia. However, there is no evidence or no conducted survey/research data or document regarding trachoma prevalence in Areka Town. We, therefore, did a study to assess the prevalence of active trachoma and associated factors in Areka Town in South Ethiopia.

**Methods:**

A community-based cross-sectional study was employed. A total of 586 children aged 1–9 years were involved. We compiled a structured questionnaire from the relevant literature and pretested before use. A range of data was collected on the sociodemographic, facility, and service-related, and environmental factors. The outcome variable was measured by using frequencies, cross-tabulation, and percent. Multivariate logistic regression was applied to control potential confounders and to identify the predictors.

**Results:**

This study revealed that 37.9% of children aged 1–9 years have active trachoma (95% CI: 34%–42%). Households without latrine (AOR = 6.88; 95% CI: 2.13–22.18), openly disposing domestically produced waste (AOR = 4.62; 95% CI: 2.41–8.83), cooking in the same room (AOR = 5.13; 95% CI: 2.21–11.88), and using the cooking room without a window (AOR = 2.28; 95% CI: 1.11–4.69) were more likely to have their children develop active trachoma. Similarly, children with caretakers having inadequate knowledge about trachoma (AOR = 8.10; 95% CI: 2.04–32.17) were more likely to develop active trachoma. However, households consuming more than 20 liters of water per day were 82% (AOR = 0.18; 95% CI: 0.07–0.44) less likely to have their children develop active trachoma while compared to those consuming less than the figure.

**Conclusions:**

The prevalence of active trachoma in the children aged 1–9 years in the study area was found to be high, and it is much higher than the WHO elimination threshold.

## 1. Introduction

Trachoma is a bacterial eye infection caused by the bacterium called *Chlamydia trachomatis* [1–3]. It can be transmitted from person to person through contact with infected ocular and nasal discharges, often through fingers and clothing or fomites, and is also spread by eye-seeking flies. In a typical endemic setting, repeated chlamydia infection of the conjunctiva begins early in life (1–2). This can initiate recurrent episodes of chronic conjunctival inflammation, characterized by the formation of lymphoid follicles [1–4].

According to the World Health Organization simplified classification or grading of trachoma infection [5, 6], follicular trachoma (TF) is described as the presence of 5 or more follicles (of at least 0.5 mm) in the upper tarsal conjunctiva, trachomatous inflammation (TI) is described as conjunctiva that obscures more than half of the deep normal tarsal vessel, trachomatous scarring (TS) is described as the presence of scarring in the tarsal conjunctiva, trachomatous trichiasis (TT) is described as at least one lash touches the eyeball, and corneal opacity (CO) is described as the presence of easily visible corneal opacity which obscures at least some of the pupil [5, 6].

Trachoma is known to be one of the major causes of blindness in Ethiopia. However, recent data that indicate the disease burden were lacking [7]. The national prevalence of active trachoma (either TF or TI) for children in the age group of 1–9 years is 40.14%. Considerable regional variations are observed in the occurrence of active trachoma; the highest prevalence is in Amhara (62.6%), Oromia (41.3%), and Southern Nations, Nationalities, and People's Region (SNNPR) (33.2%), Tigray (26.5%), Somali (22.6%), and Gambella (19.1%). The rural prevalence of active trachoma is almost fourfold compared to the urban prevalence (42.5% rural vs. 10.7% urban) [2, 5, 8]. The national prevalence of trachomatous trichiasis (TT) is 3.1% with the highest prevalence in Amhara Regional State (5.2%). Trachomatous trichiasis is higher in females compared to males (4.1% vs. 1.6%). Over 9 million 1–9-year-old children live with active trachoma [2, 5, 7, 8]

The intensive effort of the Global Elimination of Trachoma of 2020 (GET 2020) has successfully reduced the global burden of active trachoma from 84 million cases in 2003 to 21.4 million cases in 2012 [2, 5, 9, 10]. This success has been partly due to the surgery, antibiotics, facial hygiene, and environment (SAFE) strategy. However, currently, about 334, 000 disability-adjusted life years are lost due to trachoma [2, 5, 9]. In addition, blindness from trachoma and care of patients with trachoma slows down economic growth and that leads to a global productivity loss of $3 billion to $6 billion (average $ US 5.3 billion) annually. This loss is from the poorest community where trachoma is endemic such as Ethiopia [5]. About 229 million people live in endemic areas, 176 million live in Africa, and 80% of the global burden is now limited to 14 countries including Ethiopia [2, 5, 9].

According to a Ministry of Health report, the SAFE strategy has been implemented in some regions of the country, such as Amhara Region. Amhara Region can demonstrate promising success result in controlling trachoma by implementing the SAFE strategy. Likewise, 14.7 million people in the region were covered by azithromycin mass administration in 2010, and more than 89, 000 latrines were constructed in the region within one year. However, to scale up the intervention to other areas, the main problem is a lack of data. According to the International Coalition for Trachoma Control (2011) reports, about 46 million Ethiopians are living in trachoma suspected endemic areas. Recommendations of the different studies were that Ethiopia, as one of the priority countries, needs an urgent mapping of trachoma. It is important to improve the quality and availability of epidemiological data to guide the trachoma elimination program of the area [2, 5, 9].

Identifying the magnitude of trachoma and associated factors could, therefore, help to design context-specific trachoma prevention and control intervention strategies such as the SAFE strategy, and the World Health Organization (WHO) and its partners are targeting the Global Elimination of Trachoma as a cause of blindness by the year 2020 (GET2020) [2, 5, 9]. Active and potentially blinding trachoma is highly prevalent throughout SNNPR, and in 108 woredas, the disease presents a significant public health problem [5].

GET2020 trachoma prevention, control, and intervention program such as the SAFE strategy is strongly influenced by institutional factors and traditional practices in the community such as Ethiopia [5]. Some of these factors are providing traditional medicine to individuals with active trachoma signs, not taking trachoma cases to health facilities due to that they believe it as a mild and not harmful problem, believed that the child becomes healthy later; they may also believe that either there is no medicine at the health post and/or even at the health-center level [5].

Therefore, the area needs undertaking further research to identify the ground to reduce the burden of trachoma, especially at community-level that helps the government and implementing partners to make an evidence-based decision in promoting and provision of quality-care, and therefore, the purpose of this study will be to assess the prevalence and factors associated with trachoma in Areka Town.

## 2. Methods

### 2.1. Study Setting and Period

The study was conducted in Southern Nations, Nationalities, and People's Region (SNNPR), Wolaita Zone, Areka Town. It is located in 329 kilometers south of Addis Ababa, 85 kilometers from Hawassa, and 29 kilometers from the capital city of Wolaita Zone (Sodo). Areka Town is one of three city administrations (namely, Sodo Town, Areka Town, and Bodity Town) of Wolaita Zone. The study was conducted from September 01–31/2018. There are one governmental health center, one nongovernmental hospital (Dubo St. Mary Hospital), and four kebeles in Areka Town, and trachoma-related services are performed in all health settings in the study area. The town has a total population of 60827, 1–9-year-old children account for 32% (19465), and it has 6488 households.

### 2.2. Study Design

The community-based cross-sectional study was conducted in Areka Town, SNNPR.

### 2.3. Source Population

All children in Areka Town from 1–9 years of age.

### 2.4. Study Population

Children from 1–9 years of age those are selected by using a systematic sampling method.

### 2.5. Inclusion Criteria

All selected children aged 1–9 years (selected from the integrated registration book).

### 2.6. Exclusion Criteria

All children from 1–9 years of age who are unable to undergo physical examination due to serious medical illness (other than eye cases) and who live in the households with a selected child, but not selected (even if they undergo eye examination for sake of treatment).

### 2.7. Study Variables

  Outcome variable: active trachoma (presence of follicular trachoma (TF) and/or inflammatory trachoma (TI)).
  Independent variables: environmental factors (access to latrines, solid waste disposal, cooking room condition and domestic water consumption, source of water, distance to the water source, compound cleanness, and keeping animals in houses).
  Socioeconomic and demographic factors (sex, age, family size, educational status, number of 1–9 years of age children, average monthly income, and occupation).
  Individual/personal factors (clean face, knowledge of caretakers, and discharge on the ocular and nasal area).

### 2.8. Sample Size Determination

The sample size was calculated by using the single population, which was

(1) $$\begin{array}{c} n=\frac{z^{2}pq}{d^{2}}, \end{array}$$

where *n* = the desired sample size when the target population is over ten thousand people. *z* = the standard normal deviate corresponding to 95% confidence interval (*z* = 1.96). *p* = the proportion of the target population estimated to have the characteristics being investigated; hence, *p* = 33.2%, of active trachoma in SNNPR. *q* = the proportion of the target population without the characteristics being investigated (*q* = 1 − *p*). *d* = the degree of accuracy at 95% confidence interval, and let it be (0.04). Sample size *n*=([(*Zα*/2)^2^*∗p*(1 − *p*)]/*d*^2^).

By using *p* of SNNPR, the sample was found to be 532. With a 10% nonresponse rate, the total sample required was 586.

### 2.9. Sampling Technique

All four kebeles were included in the study, and the required sample size of 586 was proportionally allocated to each kebele based on their total target population (1–9 years children); finally, the study subjects and households were selected by using a systematic sampling technique (every *K*^th^ = 3).

### 2.10. Data Collection Procedures

Data were collected from September 01–31/2018 using questionnaires and an observational checklist. Structured questionnaires were prepared by the investigator, which include the basic sociodemographic, environmental, and behavioral characteristics regarding the trachoma prevalence of households in the communities of the study area. The questionnaires were originally developed in English and then translated into the local language (Amharic). The Amharic version was later translated back into English with the help of language professionals/experts. All necessary corrections were made for the actual questionnaire. The questionnaire was pretested in households of Achura Kebele which was near to the study area that had similar characteristics to the areas where the actual study was carried out.

Training on eye examination, grading, and reporting the result was given to the graders/collectors for two days. Immediately after the training, health professionals went to the field to perform eye examinations on selected study children. The guide used for reporting examination results was the simplified trachoma grading scheme, which was developed by the WHO for fieldwork. The examiner then should evert the upper eyelid to examine the conjunctiva over the stiffer part of the upper eyelid (tarsal conjunctiva). The normal conjunctiva of the upper tarsal area is pink and is smooth, thin, and transparent. Over the whole area of the tarsal conjunctiva, there are large deep-lying blood vessels that mainly run vertically from the upper and lower edges of the tarsal plate. Based on the presence or absence of signs of trachoma, cases are graded as trachomatous inflammation-follicular (TF) and trachomatous inflammation intense (TI), in children aged 1–9 years. Finally, the presence or absence of each sign of trachoma was recorded on the collection form for each study individual.

### 2.11. Operational Definitions

#### 2.11.1. Active Trachoma

Active trachoma includes either TF and/or TI seen at upper tarsal conjunctiva. It manifests with foreign body sensation, pain which is worsened during blinking, tearing, fear of light, and mild eye discharge seen in the morning.

#### 2.11.2. Household

It consists of one or more people who live in the same house.

#### 2.11.3. SAFE

It is a strategy developed to eliminate blindness caused by trachoma through doing surgery, antibiotic treatment, facial cleanliness, and improving the environment.

#### 2.11.4. Adequate Water

It is recommended a minimum average of 20 liters (1-2 pots) per person per day of water supply for all basic needs is considered adequate.

#### 2.11.5. An Improved Toilet Facility

It is a structure used by household members and able to separate waste from human contact.

#### 2.11.6. Caregivers

Caregivers are caretakers or parents of children who took care of the selected children at the time of data collection.

#### 2.11.7. Unclean Face

The presence of “sleep” (or ocular discharge) around on the eyes and the presence of nasal discharge on the upper lip or cheeks.

#### 2.11.8. Clean Face

Absence of dirt, dust, and nasal or eye discharge on cheeks and forehead.

#### 2.11.9. Serious Medical Illness

Illness other than eye case in which unable to undergo a physical examination.

#### 2.11.10. Knowledge about Trachoma

The median value of the records of the four items used to assess the knowledge of caretakers was taken. Those respondents having scored greater or equal to the median value were categorized as knowledgeable, and those who have scored less than the median were categorized as less knowledgeable.

All the above definitions were adopted from Operational Definitions for NTDs control program, Kenya University in collaboration with WHO/UNICEF, May, 2016.

### 2.12. Data Quality Management

To ensure the quality of the data, the tool was checked for face validity by use of the pretest on 29 caretakers (5%). The training was given for 4 diploma nurses who are from nearby health centers of Achura as data collectors, and for who are BSc nurses or public health officers and/or those trained on ophthalmic issues or IECW (integrated eye care worker) was used as a supervisor. The data collectors and supervisors were trained for two days on the techniques, ways of data collection, about the objectives of the study, and variables on the data abstraction sheet.

Throughout the progress of the data collection, interviewers were supervised at each site, regular meetings were held between the data collectors and the principal investigator together in which problematic issues arising from interviews which were conducted and mistakes found during editing were discussed, and decisions were reached. The collected data were reviewed and checked for completeness before data entry; the incomplete data were discarded. The data entry format template was produced and programmed. Double entry was done on 10% questionnaires to check consistency by using EpiData software. The simplified WHO trachoma grading system was used [11] to identify persons with trachoma. Both eyes were graded separately, for active trachoma in children aged 1–9 years.

### 2.13. Data Analysis Procedures

The coded data were entered into EpiData 3.1 and were exported to SPSS version 20 software package for further statistical analysis. A *P* value <0.25 was a candidate for multivariate analysis, and a *P* value <0.05 was considered significant. Using logistic regression, multivariable analysis was also carried out. The odds ratio and 95% confidence interval (CI) were used to determine the effect of potentially associated variables on household prevalence of trachoma, and multicollinearity issues were checked. The goodness of fit of the final model was checked using the Hosmer and Lemeshow test of goodness of fit considering good fit at *P* value <0.05 level of significance.

### 2.14. Ethical Consideration

Ethical clearance was obtained from the research and publication office of Wolaita Sodo University Institute of Public Health ethical review board. Permission letters were obtained from the Wolaita Zone Health Department and Areka Town Health Office. Finally, practical permission was obtained from the respective kebele. Consent was gained from each interviewee by confirming that all the data extracted will be kept confidential and will not be used for any other purposes than the stated research objective. Finally, children with active trachoma were treated according to the guideline.

## 3. Results

### 3.1. Characteristics of the Respondent Answered the Questionnaire

Five hundred eighty-six respondents participated in this study with an overall response rate of 100%. Two hundred sixty-nine (45.9%) of the study participants were females. The mean age of the respondents was 37.85 ± 7.7 SD. Five hundred eight (86.7%) of the respondents were married. One hundred seventy-six (30%) were in the age group of 35–39 years. As to their educational status, 60 (10.2%) were unable to read and write, and the rest had achieved some level of education. Concerning occupation, 223 (38.1%) of their households live on selling/buying (merchant) as their main income (Table 1).

### 3.2. Knowledge of Respondents towards Trachoma

Regarding the knowledge of respondents towards trachoma, 478 (81.7%) of respondents know what the trachoma is. Four hundred thirty-two (73.7%) of respondents answered that trachoma is a preventable disease. Four hundred twenty-eight (73%) of respondents replied that blindness can be caused by trachoma. Four hundred twenty-four (74.4%) of respondents had information that trachoma is transmittable (Table 2).

**Figure 1:**
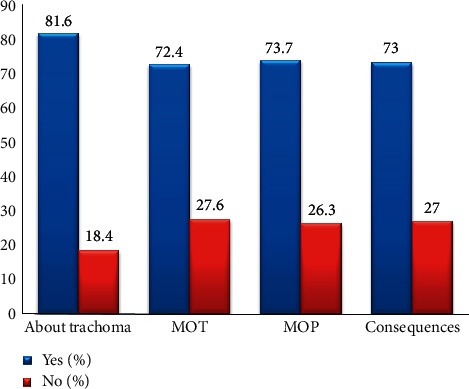
Caretakers knowledge towards Trachoma in in Areka Town in Southern Ethiopia, 2018.

The median value of the knowledge of children's caretakers was calculated as 430 (73.4%) (as shown in Figure 1).

### 3.3. The Practice of Respondents towards Trachoma Infection

**Figure 2:**
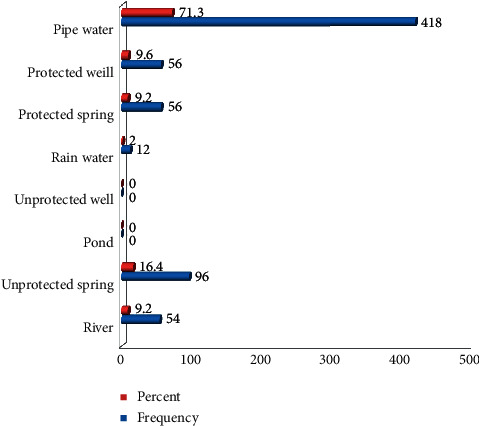
Source of water for domestic use in Areka Town in Southern Ethiopia, 2018.

In the respondents' practice towards specific dimensions, only 141 HHS (14.1%) consume less than the recommended daily adequate (20 litters) water per day with the rest 75.9% consuming the recommended amount to prevent trachoma infection. Two hundred seventy-two (46.4%) respondents travel <30 minutes to get water for domestic use. Three hundred seven HHS (52.4%) use a separate kitchen for cooking. Two hundred four (34.8%) of respondents simply discard the waste nearby the house. Regarding water source, the majority (418) of the respondents use a pipe as a water source followed by 96 of them use unprotected spring (Figure 2).

Based on this study, from 255 (43.5%) respondents simply disposing domestically of produced waste, 204 (80%) respondents discard simply near the living house. Out of 586 respondents, 418 (71.3%) respondents were using pipe water. Three hundred forty of the examined children (58%) had a relatively clean face with the rest 42% having had unclean (Table 3).

### 3.4. Factors Associated with the Prevalence of Active Trachoma

Bivariate logistic regression analysis was performed to identify predictor variables of overall trachoma prevalence. The crude odds ratio (COR) along with a 95% confidence interval was estimated to assess the association between each independent variable and the outcome variable. Variables with a *P* value <0.25 in the bivariate logistic regression analysis were considered as candidate variables for the multivariable model.

During bivariate analysis, variables that were associated with the prevalence of active trachoma were households with no latrine, households cooking in the same room, households cooking room with no window, households those simply disposing waste, households consuming less than 20 liters of water, households those were with no knowledge of trachoma prevention, households those were traveling 30–59 minutes to fetch water, households those do not know trachoma, households with no knowledge about trachoma that can cause blindness, households with no knowledge about trachoma transmission, households with cattle, and eligible children but not attending the class. However, during multivariate logistics analysis, households with no latrine, households those cook in the same room, households whose cooking room with no window, households of simply disposing of waste, households of consuming less than 20 liters of water, and households those do not know trachoma were predictors of active trachoma.

Children living in a household without latrine were approximately seven times (AOR = 6.88; 95% CI: 2.13–22.18) more likely to develop active trachoma while compared with those children who live in a household having latrine. Similarly, Children living in the household cooking in the same room were above five times (AOR = 5.13; 95% CI: 2.21–11.88) more likely to develop active trachoma while compared with children who live in households those cook in separate room/kitchen. This study also revealed that children living in a household in which the cooking room has no window were about 2 times (AOR = 2.28; 95% CI: 1.11–4.69) more likely to develop active trachoma compared to those with window. Children living in a household of openly disposing domestically of produced waste were more than 4 times (AOR = 4.62; 95% CI: 2.41–8.83) more likely to develop active trachoma while compared to those children living in a household that bury/burn the waste. Children living in a household that consume more than 80 liters of water were 93% (AOR = 0.07; 95% CI: 0.01–0.39) less likely to develop active trachoma when compared to those who consume less than 80 liters per day. This study also depicts that respondents who do not know what trachoma were about 8 times (AOR = 8.10; 95% CI: 2.04–32.17) more likely to have their children affected by active trachoma while compared with respondents know well about trachoma (Table 4).

## 4. Discussion

Active trachoma infection might have its own effect on contributing to a high burden of blindness among children aged 1–9 years. The primary aim of this study was to assess the prevalence of active trachoma on 1–9 years of age children and its predictors in Areka Town in South Ethiopia.

The overall prevalence of active trachoma of 1–9-year-old children was 37.9% (95% CI: 34–42%). The proportion of active trachoma in the current finding was lower than the result in Amhara (62.6%) [7], Amaro and Burji woredas (48.5%) [12], and Horo Guduru Woreda (48.9%) [13]. The difference might be due to the difference in sample size and study population. However, it was higher than other related studies conducted in different parts of Ethiopia: Baso Liben District (24.1%) [14], Oromia Region (23.4%) [13], Amhara (21.6%) [5], Somali (22.6%), SNNPR (33.2%), Tigray (26.5%), and Gambella (19.1%) [7], and Dera Woreda (15.6%) [15], Dembia District (18%) [3], South Wollo (26.4%) [16], and Harari Regional State (1.3%) [17]. It was also higher than related studies from Senegal (2.5%) [8], Nigeria (0.04–19%) [18], Kenya (18.7%) [2], and Burkina Faso (9.5%) [19]. The difference might be due to the difference in sample size and study population, and the disparities in the socioeconomic status of the respondents. However, the current finding is somewhat similar to the result of studies conducted at the national level (40.14%), Oromia (41.3%), Zala District of Gamo Gofa Zone (36.7%) [20], and Senegal (38%) [8].

In this study, about 88% (517) respondents have access to a latrine, and the regression analysis showed that inadequate access to latrine was significantly and positively associated with the prevalence of active trachoma (AOR = 6.88; 95% CI: 2.13–22.18). The study of Beselam Tadesse and three other researchers in the North and South Wollo Zones of Ethiopia revealed that having access to latrine was negatively and independently associated with active trachoma prevalence [5]. This result shows a similarity with the current study finding when interpreted in another way. The current finding is also similar to other studies conducted in Dera Woreda [15], Girar Jarso Woreda [4], Somali Region [9], SNNPR [12], and different parts of Ethiopia [18, 21].

In the current study, out of total 586 households interviewed, more than 52% (307) of the households use a separate kitchen for cooking and the rest 47% (279) cook in the same house. Three hundred eighty-two (65.2%) cooking rooms have a window, and the remaining 204 (34.8%) do not have a window. Finding from this study revealed that cooking in the same house/room (AOR = 5.13; 95% CI: 2.21–11.88) and those cooking rooms without window (AOR = 2.28; 95% CI: 1.11–4.69) made significant association with the prevalence of active trachoma while compared with using separate room/kitchen for cooking, and cooking in a kitchen having a window, respectively. No research findings depicted a similar outcome in these particular variables, so that this particular study result contradicts to other research studies, and might need further investigation by another scholar.

In this particular study, openly disposing of domestically produced wastes was positively and significantly associated with developing active trachoma (AOR = 4.62; 95% CI: 2.41–8.83). Many other studies also showed similar results in Girar Jarso, Dembia and Dera, Mojo, and Lume Woredas of Ethiopia [3, 4, 15, 21], and Senegal [8]. Moreover, a study conducted in Harari Region showed that the collection of wastes by the municipality after households' openly disposing of domestically produced wastes was significantly associated with active trachoma [22]. Indeed, many types of research studies that were done in different parts depicted that domestically produced waste disposal have no appreciable association with the prevalence of active trachoma [5, 9, 10, 13, 19, 21, 23]. This is controversial to the current study.

In the current study, 24.1%, 45.9%, 21.8%, and 8.2% of the households consume less than 20 liters, 20 to 40 liters, 40 to 80, and more than 80 liters of water on a daily base, respectively. This study showed that households' daily water consumption of more than 20 liters was negatively and notably associated with developing active trachoma (AOR = 0.18; 95% CI: 0.07–0.44). This result was similar to the other study conducted in Girar Jarso Woreda of North Shewa and Baso Liben District of East Gojam which revealed that those households consuming less than 20 liters of water per day were more likely to achieve active trachoma [4, 14]. However, many study results conducted in different parts of the country revealed that domestic water consumption in daily base did not make a significant connection with the development of active trachoma [3, 5, 8, 9, 13, 15–17, 21, 24, 25].

In the current study, about 430 (73.4%) of the respondents had adequate knowledge about trachoma, with the rest 156 (26.6%) having had inadequate knowledge. This finding depicted that less knowledge about trachoma as a disease was significantly and positively associated with developing active trachoma (AOR = 8.10; 95% CI: 2.04–32.17). The current finding is similar to other studies done in Baso Liben District of East Gojam [14] and Zala District of Gamo Gofa Zone [20].

## 5. Conclusion

The prevalence of active trachoma in the children aged 1–9 years studied in Areka Town, Southern Ethiopia, was found to be high, and it is much higher than the WHO elimination threshold. Children from households with less access to a latrine, openly disposing of domestically produced wastes and daily water consumption of fewer than 20 liters, and children whose caretakers have inadequate knowledge about trachoma were more likely to be affected by active trachoma. Intervention modalities that would address the identified risk factors are highly recommended to prevent and control active trachoma in this setting.

### 5.1. Recommendation

The prevalence of active trachoma in the study area is far from the elimination of trachoma as a public health problem. Thus, its prevalence can be controlled in the study area by the following:

- Improving awareness of the community is a need through health education programs regarding proper solid waste disposal using a multidisciplinary approach (offices of municipality, health, and administration of respective kebeles)
- The health sector needs to promote achieving high coverage and appropriate utilization of latrine
- The use of a separate kitchen and a cooking room with a window is needed by using a multidisciplinary approach
- Multisectorial collaboration (offices of water, health, and mayor) is needed to promote adequate recommended daily water consumption by making safe water available and accessible to the community

## Figures and Tables

**Table 1:** Sociodemographic characteristics of respondents in Areka Town in Southern Ethiopia, 2018 (*N* = 586).

Variable	Category	Frequency	Percent
Sex	Male	317	54.1
	Female	269	45.9
Age of respondents	<25 years	28	4.8
	25–29 years	46	7.8
	30–34 years	96	16.4
	35–39 years	176	30
	40–44 years	147	25.1
	45 and above	93	15.9
Marital status of respondents	Married	508	86.7
	Divorced	24	4.1
	Widowed	54	9.2
Occupation of respondents	Farmer	69	11.8
	Craftsmen	30	5.1
	Merchant	223	38.1
	Employee	180	30.7
	Housewife	75	12.8
	Retired	9	1.5
Average monthly income	≤1500 birr	245	41.8
	≥1501 birr	341	58.2
Family size	Less than 5	431	73.5
	5 and above	155	26.5
Age of observed child	Aged 1–4 years	316	53.9
	Aged 5–9 years	270	46.1
No. of <9-year-old child	2 or less	547	93.3
	3 and above	39	6.7
Educational/enrollment status of observed children	Not eligible	322	54.9
	Eligible, not enrolled	39	6.7
	Enrolled	225	38.4

**Table 2:** Knowledge of respondents and source of information towards trachoma in Areka Town in Southern Ethiopia, 2018 (*N* = 586).

Variable	Category	Frequency	Percent
Do you know what the trachoma is?	Yes	478	81.7
	No	108	18.4
Do you know that blindness is caused by trachoma?	Yes	428	73
	No	158	27
*Do you think that trachoma is transmittable?*	Yes	424	72.4
	No	162	27.6
Is trachoma transmitted through flies?	Yes	409	69.8
	No	15	2.6
Is trachoma transmitted through contaminated fingers?	Yes	199	34
	No	225	38.4
Is trachoma transmitted by sharing contaminated towels?	Yes	108	18.4
	No	316	27.6
*Can we prevent trachoma?*	Yes	432	73.7
	No	154	26.3
Can we prevent trachoma by burying/burning wastes?	Yes	179	30.5
	No	251	42.8
Can we prevent trachoma by treating cases?	Yes	172	29.4
	No	258	44
Can we prevent trachoma by separating cattle shelter from human living?	Yes	66	11.3
	No	364	62.1
Can we prevent trachoma by using separate kitchen?	Yes	75	12.8
	No	355	60.6
Is information about trachoma got from health personnel?	Yes	391	66.7
	No	42	7.2
Is information about trachoma got from mass media?	Yes	141	24.1
	No	292	49.8
Is information about trachoma got from others/neighbors?	Yes	229	39.1
	No	204	34.8

**Table 3:** Practice of respondents towards trachoma in Areka Town in Southern Ethiopia, 2018 (*N* = 586).

Variable	Category	Frequency	Percent
Households' daily water consumption	<20 liters	141	24.1
	20–40 liters	269	45.9
	40–80 liters	128	21.8
	80 and more	48	8.2
Source of water	River	54	9.2
	Unprotected spring	96	16.4
	Rainwater	12	2
	Protected spring	56	9.2
	Protected well	56	9.6
	Pipe water	418	71.3
Traveling the distance to get water	In a compound	200	34.1
	<30 minutes	272	46.4
	30–59 minutes	114	19.5
Place of cooking	In the same room where they sleep	141	24.1
	In the same house but a different room	138	23.5
	In the separate kitchen	307	52.4
Does your cooking room have a window?	Yes	382	65.2
	No	204	34.8
What do you do the produced waste?	Bury/burn	331	56.5
	Discard	255	43.5
If simply discard, where do you dispose of the waste?	Nearby the house	204	34.8
	Far away from the house	45	7.7
Do you have a latrine?	Yes	517	88.2
	No	69	11.8
If you have a latrine, what type?	Covered pit latrine	219	37.4
	Uncovered pit latrine	274	46.8
	VIP latrine	21	3.6
	Latrine with water carriage system	3	0.5
Do you have your own cattle?	Yes	245	41.8
	No	341	58.2
If you have cattle, where do you pass/integrate them in the night?	In the same house	129	22
	In the same house in a different room	57	9.7
	In separate shelter	53	9
Facial cleanliness of children	Clean	340	58
	Unclean	246	42

Out of 586 children who were examined for active trachoma, 222 (37.9%) of children developed active trachoma and the rest, 364 (62.1%) children, were negative for active trachoma.

**Table 4:** Factors associated with prevalence of active trachoma in Areka Town in Southern Ethiopia, 2018 (*N* = 586).

Variable	Category	Total children	Children with active trachoma	*P* value	COR (95% CI)	PV. adj.	AOR (95% CI)
		No	No				
Do you have a latrine?	Yes	517	159		1		1
	No	69	63	0	23.6 (10.03–55.76)	<0.01	6.88 (2.13–22.18)
Place of cooking	In the same room	141	17	0	28.38 (16.52–48.76)	0	5.13 (2.21–11.88)
	The same house but different room	138	60	0	4.48 (2.82–7.10)	0.68	0.86 (0.42–1.76)
	In separate kitchen	307	45		1		1
Does your cooking room have a window?	Yes	382	72		1		1
	No	204	150	0	11.96 (7.99–17.90)	0.02	2.28 (1.11–4.69)
Domestically produced waste disposal	Bury/burn	331	42		1		1
	Simple disposal	255	180	0	16.51 (10.84–25.16)	0	4.62 (2.41–8.83)
Water consumption per day	Less than 20 liters	141	111		1		1
	20–40 liters	269	84	0	0.12 (0.08–0.20)	0	0.18 (0.07–0.44)
	40–80 liters	128	24	0	0.06 (0.03–0.11)	0.06	0.38 (0.14–1.05)
	More than 80 liters	48	3	0	0.02 (0.01–0.06)	<0.01	0.07 (0.01–0.39)
Knowledge	Knowledgeable	430	90		1		1
	Less knowledgeable	156	132	0	20.78 (12.69–34.02)	0.42	1.83 (0.42–7.92)
Traveling the distance to fetch water	In a compound	200	36		1		1
	Less than 30 minutes	272	99	0	2.61 (1.68–4.04)	0.89	0.96 (0.53–1.75)
	30 to 59 minutes	114	87	0	14.68 (8.36–25.77)	0.11	0.37 (0.11–1.24)
Know about what trachoma is	Yes	478	123		1		1
	No	108	99	0	31.75 (15.57–64.74)	<0.01	8.10 (2.04–32.17)
Know that trachoma causes blindness	Yes	425	96		1		1
	No	161	126	0	12.34 (7.96–19.12)	0.18	0.50 (0.18–1.37)
Knowledge about the communicability of trachoma	Yes	424	84		1		1
	No	162	138	0	23.27 (14.19–38.18)	0.35	1.96 (0.49–7.86)
Owner of cattle?	Yes	245	153	0	6.56 (4.53–9.49)	0.19	1.49 (0.82–2.69)
	No	341	69		1		1
Educational status of the child	Not eligible	322	13		1		1
	Eligible but not attending	39	33	0	8.9 (4.53–9.49)	0.45	1.74 (0.41–7.37)
	Enrolled	225	66	0.03	0.67 (0.47–0.96)	0.89	1.69 (0.92–3.12)

## Data Availability

The datasets used and/or analyzed during the current study are available from the corresponding author on reasonable request.

## Abbreviations

CHWs: Community health workers
CO: Corneal opacity
GET: Global Elimination of Trachoma
GET2020: WHO Alliance for the Global Elimination of Trachoma by the year 2020
ITI: International Trachoma Initiative
MOH: Ministry of Health
MOPHS: Ministry of Public Health and Sanitation
MOP: Mode of prevention
MOT: Mode of transmission
SAFE: Surgery, antibiotics, facial hygiene, and environment
SNNPR: Southern Nations, Nationalities, and Peoples' Region
TF: Trachoma follicular
TF: Trachomatous follicular
TS: Trachomatous scarring
TT: Trachomatous trichiasis
WASH: Water, sanitation, and hygiene.
